# Identification of Additional Trials in Prospective Trial Registers for Cochrane Systematic Reviews

**DOI:** 10.1371/journal.pone.0042812

**Published:** 2012-08-15

**Authors:** Wynanda A. van Enst, Rob J. P. M. Scholten, Lotty Hooft

**Affiliations:** Dutch Cochrane Centre, Department of Clinical Epidemiology, Biostatistics and Bioinformatics, Academic Medical Center, Amsterdam, The Netherlands; Johns Hopkins Bloomberg School of Public Health, United States of America

## Abstract

**Background:**

Publication and selective outcome reporting bias are a threat to the validity of systematic reviews. Extensive searching for additional trials in prospective trial registers could reduce this problem. We have evaluated how authors of Cochrane systematic reviews currently make use of trial registers as an additional source for the identification of potentially eligible trials.

**Methodology/Principal Findings:**

We included 210 systematic Cochrane reviews of interventions published between 2008 and 2010 of which the protocol was first published in 2008. When prospective trial registers were searched we recorded the names of the register(s), the authors' motive(s) and if they yielded any extra trials.

In 80 reviews (38.1%) the authors had searched in one or more prospective trial register(s) of which 55% had searched in overlapping search portals and individual registers. Most frequently assessed were the MetaRegister (66.3%) and Clinicaltrials.gov (60%) which is in sharp contrast of other registers or portals like the WHO ICTRP Search Portal (20%). Reported motives to use registers were to identify ongoing trials (83.3%), to identify unpublished outcomes or trials (23.5%), to identify recently published trials (11.8%), or to identify any relevant trial (3.9%).In 28 reviews (35%) the authors had selected (ongoing) trials identified in trial registers as potentially eligible.

**Discussion:**

Trial registers as an additional source of information are gaining acknowledgement amongst Cochrane reviewers. Nevertheless, searches seem to be inefficient as overlapping databases are frequently consulted, while the WHO ICTRP Search Portal that includes the data from all approved registers worldwide is being underused. Moreover, the emphasis is now on the identification of ongoing trials, although the prospective registers offer a broader potential. Further familiarity of registers and guidance how to search and to report will help to implement this as a common method and utilize the full potential of prospective trial registers for systematic reviews.

## Introduction

Systematic reviews of randomized controlled trials (RCTs) are regarded as the highest level of evidence to guide decisions in healthcare. Cochrane reviews in particular are of high quality because these reviews follow explicit, transparent and systematic methods [1], [2]. The results of systematic reviews, however, can be biased when the included evidence does not offer a fair representation of all existing evidence. Empirical evidence consistently suggests that statistically significant and positive findings are more likely to be published than non-significant or negative findings [3], [4] and will take shorter time to be submitted and to get published after completion of the study [5]–[8]. When publication of trials or outcomes depends on the results, publication bias and selective outcome reporting bias may arise. This can affect the results from the meta-analysis of the review and possibly also the results of a review without any meta-analysis [9].

To minimize the effects of publication bias and outcome reporting bias review authors should perform a comprehensive search to identify all relevant trials [10], [11]. Most trials can be identified in well known biomedical databases like MEDLINE or EMBASE. Nevertheless, some trials can only be identified by the use of additional strategies like contacting experts, checking the reference lists of eligible trials, handsearching of conference proceedings, searching the Internet with web search engines like Google or searching the websites of relevant organisations [12]. These strategies may be very time consuming and still do not guarantee that all relevant trials will be found.

Recently searching in prospective trial registers can be added as another strategy to identify relevant trials. Already in 1986 it was suggested that prospective registration of trials could reduce or even resolve the problems resulting from publication bias and outcome reporting bias [11], [13], [14]. However, for a long period of time trials were not systematically registered. In September 2004, however, the International Committee of Medical Journal Editors (ICMJE) announced that they would only accept manuscripts for publication as of September 2005 if essential information about the underlying trial design had been deposited into an accepted prospective trial register before enrolment of the first patient. In November 2004, the World Health Organization (WHO) was asked by the international scientific and political community to facilitate the establishment of a network of these national clinical trials registers and to develop strict criteria for ‘registry approval’ concerning the content, quality and accessibility [15]. Currently, there are 15 registries that meet these strict international requirements [16].

The prospective registration policy of the ICMJE was adopted by many biomedical journals. Trial registration has become common and the number of registered trials has grown considerably [17]. Authors can search in the prospective trial registers for ongoing trials, for completed trials that have not published the results (yet) or to check whether the primary outcome has changed or if all outcomes have been reported. The various national or regional trial registers can be searched individually or simultaneously through search portals that include various other registers e.g. the WHO International Clinical Trial Registry Platform (ICTRP) and the MetaRegister of Current Controlled Trials.

Our objective was to evaluate how authors of Cochrane systematic reviews make use of trial registers as an additional source for the identification of potentially eligible trials.

## Methods

### Selection of reviews

For this study, we included reviews with a protocol published in 2008 that had been converted into a full Cochrane Review by February 2010. The Cochrane Collaboration's Information Management System (ARCHIE at archie.cochrane.org) was searched to identify all Cochrane protocols that were published in 2008 and the Database of Systematic Reviews was searched for full Cochrane reviews in February 2010. Cochrane Diagnostic Test Accuracy reviews, Cochrane Methodology reviews and Cochrane Overviews of reviews were excluded.

The publication date of protocols (2008) was chosen, because the Cochrane Handbook for Systematic Reviews of Interventions which authors are required to read and follow as a guide had added the statement that: ‘Trial registers, are the best solution to unpublished trials and the conduct of all systematic reviews should be much simplified when the use of registers becomes widespread’ at the end of 2006 [18].

### Data extraction

We extracted information on three subjects. First, of each included review we extracted the applied strategies for the identification of additional potentially eligible trials and emphasized on the use of prospective trial registers. We distinguished between three methods to assess trials in a prospective trial registers:

1. search portals with which one can search in various trial registers,
2. national or regional registers that are approved by the ICMJE or WHO,
3. non-approved registers (e.g. registers of the pharmaceutical industry, non-approved national registers or registers of specialized foundations).

Second, in case any of these three methods was applied, we searched the review for a particular motive the author had for searching in the prospective trial registers. These motives were retrospectively classified as identification of ongoing trials, identification of unpublished trials or outcomes, identification of recently completed yet unpublished trials, or identification of any relevant trial.

Third, for every review for which prospective trial registers were searched, we registered if the searches had yielded trials from prospective trial registers. We classified trials as identified in a prospective trial register when the trial identification number was reported or when the reference of the trial included a link or a reference to a prospective trial register without any other reference to a publication in a journal. Cochrane reviews distinguish among four types of references: included studies, excluded studies, ongoing studies and studies awaiting assessment. The number of reviews that had identified trials from prospective trial registers was registered for each type of reference.

Two reviewers independently extracted data. Disagreements were resolved by discussion and in case of persistent disagreement a third expert was asked to make a decision.

## Results

We identified 519 protocols for Cochrane reviews that were published in 2008. Of these protocols 212 were converted into a full systematic review in the Cochrane Database of Systematic Reviews by February 2010. Two reviews were excluded because they were Diagnostic Test Accuracy reviews. The final set of systematic reviews consisted of 210 Cochrane reviews. These reviews were published in 2008 (n = 7), 2009 (n = 147) and 2010 (n = 56).

Most applied strategies to identify additional potentially eligible trials were checking the reference lists (83.3%) and contacting experts (49.0%) (table 1). In 80 of the included reviews (38.1%), the authors had searched in at least one prospective trial register either by using a search portal, a national or regional register approved by the ICMJE or WHO, or a non-approved register. Of those 80 reviews the MetaRegister of Current Controlled Trials was the most frequently used search portal (66.3%) and the WHO ICTRP search portal was used in only 20.0% (table 2). Clinicaltrials.gov was the most searched individual register (60.0%) which is in sharp contrast with other registers (table 2). In 75 reviews (93.8%) the authors had searched in a search portal or register that is approved by the ICMJE or WHO, leaving 5 reviews in which only non-approved registers were assessed.

**Table 1:** Methods applied for identification of trials in addition to searching in biomedical databases in 210 Cochrane reviews.

Method	Number of reviews (%)*
Checking reference lists	175 (83.3%)
Contacting experts	103 (49.0%)
Searching in prospective trial registers	80 (38.1%)
Handsearching of conference abstracts	78 (37.1%)
Searching the Internet	11 (5.2%)
No additional methods applied	10 (4.8%)

* Most review authors applied multiple strategies to identify additional trials. Therefore, the summation of percentages exceeds 100%.

**Table 2:** Overview of trial registers that were searched in 80 Cochrane reviews.

Type of register	Number of reviews (%)*
Search portals (all)	56 (70%)
MetaRegister of Current Controlled Trials	53 (66.3%)
WHO ICTRP Search Portal	16 (20.0%)
International Federation of Pharmaceutical Manufacturers & Associations (IFPMA)	0 (0%)
Registers approved by the WHO or ICMJE (all)	52 (65%)
Clinicaltrials.gov	48 (60.0%)
Australian New Zealand Clinical Trials Registry (ANZCTR)	8 (10%)
International Standard Randomised Controlled Trial Number Register (ISRCTN)	4 (5%)
Netherlands Trial Register (NTR)	3 (3.8%)
Chinese Clinical Trial Register (ChiCTR)	2 (2.5%)
Japan Primary Registries Network	1 (1.3%)
Non-approved registers	44 (55%)

* Most review authors searched in more than one register. Therefore, the summation of percentages exceeds 100%.

The combinations of usage are presented in table 3. In 44 reviews (55%) both a search portal and one or more individual trial registers that are already included in the search portal had been consulted, ignoring the overlap.

**Table 3:** Overview of combinations of trial registers/search portals that were searched in 80 Cochrane reviews.

Combination of usage	Number of reviews (%)
Portal only	12 (15%)
Portal and approved register	13 (16.3%)
Portal and non-approved register	11 (13.7%)
Approved register only	11 (13.7%)
Approved register and non-approved register	8 (10.0%)
Non-approved register only	5 (6.2%)
Combination of all (portal, approved register and unapproved register)	20 (25.0%)
Search strategy assessed overlapping portal and register	44 (55%)

In 51 of the 80 reviews (63.8%) one or more motives for searching in prospective trial registers were reported. Of the 51 reviews the motives were to identify ongoing trials (83.3%), to identify unpublished outcomes or trials (23.5%) (they either searched for unpublished outcomes (9.8%), unpublished trials (11.8%), or both (1.9%)), to identify recently published trials (11.8%), and to identify any relevant trial (3.9%).

In 28 of the 80 in which a search portal or register was used reviews (35.0%) the authors had yielded potentially eligible trials from a prospective trial register: in 4 reviews (14.3%) trials were actually included in the review, in 8 reviews (28.6%) the potentially eligible trials ended in the excluded category, in 20 reviews (71.4%) in the ongoing studies category and in 4 reviews (14.3%) in the category of studies awaiting classification (the total percentage exceeds 100% because some reviews found trials for multiple categories). In 34 there were no trials from prospective trial registers mentioned in the reference lists. Additionally, in 18 of the 80 reviews (22.5%) the results from extended strategies were some what confusingly documented such that we were not sure whether the reviewer had or had not identified the trial in a prospective register. None of the reviews explored the possible impact of publication bias.

## Discussion

This study indicates that the majority of Cochrane authors tried to identify additional trials through extended search strategies. In 38.1% of the reviews this extended search involves consulting any prospective trial registers. This number is a good start but should be improved in the coming years. The emphasis of the use of prospective trial registers is now on the identification of ongoing studies, but this could be much more extensive, for example to compare the outcomes of the protocol to the outcomes in the publication.

The proportion of authors that had searched in prospective trial registers to identify additional trials is promising since trial registration and its possibility to minimize bias in systematic reviews has only received major attention since 2005. In this year the ICMJE required trials to be registered in publicly accessible databases. However, compared to other strategies, like contacting experts or checking the reference lists of eligible trials, this source seems to be underused and there still seems to be room for improvement. First, search portals can be seen as the most efficient way to identify trials as these searches in multiple register at once. In our evaluation we found that most authors searched the MetaRegister of Current Controlled Trials although the WHO ICTRP Search Portal has more underlying registers it was only consulted by 20% of the review authors. Furthermore, we found that many authors consult Clinicaltrials.gov which is also accessible through both the WHO ICTRP Search Portal and the MetaRegister. The popularity of the MetaRegister and Clinicaltrials.gov may be the consequence of the guidance of the Cochrane Handbook of 2006 which emphasized on consulting this portal and register [18]. Currently, many more registers are mentioned in the Handbook, but clear guidance on which search methods are most efficient is still lacking [19]. Second, in 55% of the searches in prospective registers, redundant work was performed by searching a portal and a register that is already incorporated by the portal. This could be the results of a lack of knowledge amongst review authors but it could also be an indication that authors have doubts about the sensitivity of a search in a search portal. Especially in more extensive and complex search strategies a search in a portal could miss studies from underlying registers and the reverse [20]. Moreover, some search portals update the registered trials only weekly or monthly [21]. To ensure completeness of the search review authors might have decided to search all sources and ignore their overlap. Future improvement of the search options and more frequent updating of the various registers could make the search portals more efficient. Finally, approved registers were used in 52 reviews, whereof only three non-western registers (not American, Australian or European). Authors should actively try to search in all registers to prevent a geographical skewed distribution of trials. The WHO ICTRP Search Portal is helpful for this purpose as it searches in western and non-western registers.

The main motive for searching in prospective trial registers was to identify ongoing trials. This is not surprising because the Cochrane Handbook for Systematic Reviews of Interventions recommends consulting prospective registers for the identification of ongoing trials [22]. However, some authors seem to work ahead of guidance and had broader purposes like identification of unpublished trials or unpublished outcomes which enable controlling for or assessing publication bias or selective outcome reporting bias. This strong feature of trial registers seems to be used only occasionally and deserves more emphasis and guidance. In addition to this subject, prospective trial registers can also be consulted to compare the primary outcomes as stated in the register to the actual published primary outcomes [23]. However, this strong feature of trial registers seems to be used only occasionally and deserves more emphasis and guidance. On the other hand, to improve this feature, the quality of trial data provided in trial registers has deficiencies and needs to be improved [24]. The approved registers are most appropriate to compare the outcomes as they fulfil strict criteria on reporting. The approved registers can easily be assessed by using the WHO ICTRP Search Portal that incorporates all approved registers.

Searching in prospective trial registers seems to be worthwhile. In 35 reviews (43.8%), at least one or more trials had been identified in a trial register as potentially eligible for the review. Most of those were included in the ongoing trials section. This may alert readers and enable them to track down such trials and update the results of the reviews for their own purposes. This also applies to trials identified in trial registers that were listed in the excluded studies section. Those trials might still be important for the reader if their study question differs from the study questions of the review. Therefore, searching prospective trial registers can help to identify relevant outcomes or trials for the reviews and contribute to the completeness of the evidence and quality of the review.

Our study has some limitations. First, we studied a cohort of Cochrane reviews which apply uniform methods and include detailed reports of the results. We assume that our results do not apply to non-Cochrane reviews. Secondly, although Cochrane authors follow strict methodological and reporting criteria, it could be that not all our items of interest were transparently reported in the review. For example, according to our data, checking the reference list occurred in 83% of the reviews. This seems low for Cochrane reviews where it is standard methodology. This implies that also other items, for example the use of prospective trial registers, could have been not transparently reported, thereby underestimating the results. Incomplete reporting can also apply for the reported motives. Finally the yield from searches in prospective trial registers was poorly and inconsistent documented in almost a quarter (22.5%) of the reviews that had consulted prospective registers or search portals. Therefore, the yield of trials retrieved from prospective trial registers in Cochrane reviews is possibly underestimated. Third, it would be very interesting to measure the effect of the inclusion of trials from prospective trial registers on the results of the review but unfortunately we had too low power (n = 4) to perform sensible analysis [25], [26]. Future research should try to measure this effect.

Our study indicates that many Cochrane authors did search in prospective trial registers which has led to the identification of relevant trials for the review. However, there seems to be room for improvement. More reviewers should search prospective trial registers and search more efficiently utilizing the full potential of prospective registers instead of focussing on identification of ongoing trials. The Cochrane Collaboration should promote the use of prospective trial registers more intensively and give more guidance to authors to increase the frequency of using prospective registers. This especially applies to the usefulness of trial registers beyond the identification of ongoing trials and to the efficiency to search the WHO ICTRP Search Portal that includes all approved national or regional registers. Coordinators of prospective trial registers and search portals could help authors and trials search coordinators of Cochrane Review Groups to make their search portals more user-friendly. These measures may ensure more frequent and efficient use of current search portals and prospective trial registers using all its potential with the ultimate goal restricting biased trial results and thereby improving evidence-based decisions in healthcare.
